# Prospective Comparison of 18-FDG PET/CT and Whole-Body MRI with Diffusion-Weighted Imaging in the Evaluation of Treatment Response of Multiple Myeloma Patients Eligible for Autologous Stem Cell Transplant

**DOI:** 10.3390/cancers13081938

**Published:** 2021-04-16

**Authors:** Charles Mesguich, Valérie Latrabe, Cyrille Hulin, Axelle Lascaux, Laurence Bordenave, Elif Hindié, Gerald Marit

**Affiliations:** 1 Nuclear Medicine Department, CHU Bordeaux, F-33000 Bordeaux, France; laurence.bordenave@chu-bordeaux.fr (L.B.); elif.hindie@chu-bordeaux.fr (E.H.); 2 INSERM U1035, University of Bordeaux, F-33000 Bordeaux, France; gerald.marit@u-bordeaux.fr; 3 Radiology Department, CHU Bordeaux, F-33000 Bordeaux, France; valerie.latrabe@chu-bordeaux.fr; 4 Haematology Department, CHU Bordeaux, F-33000 Bordeaux, France; cyrille.hulin@chu-bordeaux.fr (C.H.); axelle.lascaux@chu-bordeaux.fr (A.L.)

**Keywords:** multiple myeloma, FDG-PET/CT, MRI, DWI, prognostic

## Abstract

**Simple Summary:**

Over the past decade, the imaging of multiple myeloma has evolved with the increasing use of modern imaging techniques such as 18-FDG PET/CT and MRI. Both imaging modalities have high sensitivity for the detection of bone involvement. 18-FDG PET/CT may help assess treatment response, but conventional MRI lacks prognostic value. Diffusion-Weighted is a newer MRI technique that could improve the assessment of tumor response. The aim of this prospective study was to compare the prognostic value of 18-FDG PET/CT and whole-body diffusion-weighted MRI in the evaluation of the treatment response of multiple myeloma after induction chemotherapy and after transplant. After a median follow-up of 32 months in 30 patients, we found that only 18-FDG PET/CT had significant prognostic value at two important time points of patients’ treatment. Integrating PET/CT metabolic response to the overall monitoring of disease activity might be of value in further improving patient’s management in multiple myeloma.

**Abstract:**

To compare the prognostic values of 18-FDG PET/CT (FDG-PET) and Whole-Body MRI with Diffusion-Weighted Imaging (WB-DW-MRI) in the evaluation of treatment response of Multiple Myeloma (MM) patients eligible for ASCT. Thirty patients with newly diagnosed MM prospectively underwent FDG-PET and WB-DW-MRI at baseline, after induction chemotherapy and after ASCT. Response on WB-DW-MRI was evaluated with the MY-RADS criteria. FDG-PET was considered positive if residual uptake was superior to liver uptake. Imaging results were not used for treatment modification. The impact of imaging results on PFS was analyzed. After a median follow-up of 32 months, 10 patients relapsed. With WB-DW-MRI, post-induction examination was positive in 3/25 and post-ASCT examination was positive in 3/27 patients. However, neither study showed prognostic impact on PFS. FDG-PET was positive in 5/22 post-induction and 3/26 patients post-ASCT, respectively. Positivity of FDG-PET, post-induction or post-ASCT, was associated with a shorter PFS (post-induction: median PFS 19 months vs. not reached, log-rank *p* = 0.0089; post-ASCT: median PFS 18 months vs. not reached, log-rank *p* = 0.0005). Preliminary results from this small, single-center, prospective study show that, whether performed post-induction or post-ASCT, FDG-PET has a higher prognostic value than WB-DW-MRI for treatment response evaluation of newly diagnosed MM.

## 1. Introduction

Over the past decade, the imaging of Multiple Myeloma (MM) has evolved with the increasing use of modern imaging techniques over radiographic skeletal survey (RSS). 18F-fluorodeoxyglucose positron emission tomography with computed tomography (18-FDG PET/CT) and magnetic resonance imaging (MRI) have greater sensitivity than RSS for the detection of bone involvement and are now integrated with the guidelines for the staging of MM patients [1,2,3]. Several studies have demonstrated the prognostic role of 18-FDG PET/CT performed during the monitoring of MM patients’ response to treatment [4,5,6]. Although conventional MRI is the gold standard for bone marrow imaging, it lacks prognostic value in the evaluation of MM response to treatment due to a high incidence of false-positive results [5,6,7,8]. Functional MRI techniques, such as Diffusion-Weighted imaging (DWI), may help improve the assessment of MM lesions response to treatment especially with the use of the Apparent Diffusion Coefficient (ADC) as a quantifiable biomarker of cell density [9,10,11]. Recent imaging guidelines have incorporated DWI as part of the MRI criteria for MM response assessment [12]. However, there is a lack of data regarding the prospective comparison of value 18-FDG PET/CT and Whole-Body MRI with diffusion-weighted imaging (WB-DW-MRI) in the evaluation of MM response to therapy. Therefore, we aimed to prospectively compare the prognostic value of 18-FDG PET/CT and WB-DW-MRI during the treatment response evaluation of MM patients eligible for autologous stem cell transplant response (ASCT) after induction chemotherapy and after consolidation chemotherapy post-ASCT.

## 2. Results

### 2.1. Patients Characteristics

**Figure 1:**
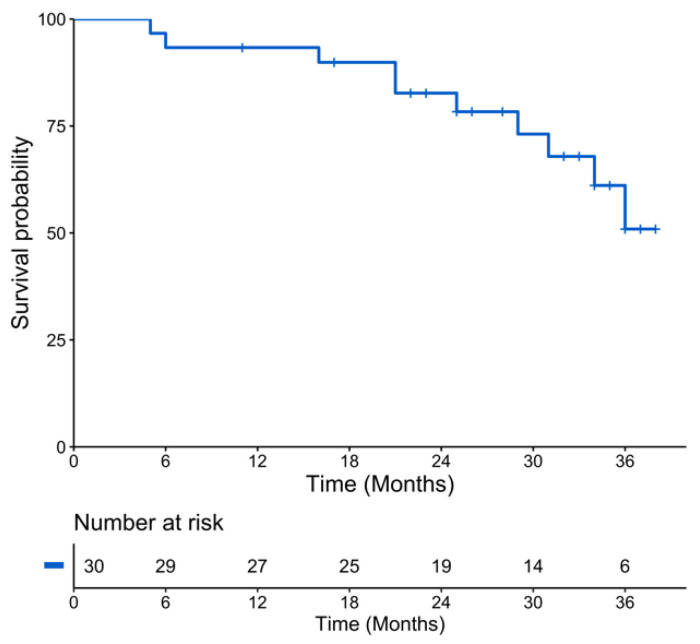
Kaplan-Meier plot of progression-free survival of the population.

Thirty consecutive patients were included in the analysis. The median follow-up was 32 months (Q1–Q3: 23–35 months). Median age was 56 years (range: 34–66 years), with 17/30 (57%) patients with an International Staging System (ISS) score of 1, 8 with an ISS of 2 and 5 with an ISS of 3. The Revised ISS (R-ISS) score was 1 in eight patients, 2 in twenty-one patients and 3 in one patient. All patients underwent induction chemotherapy, which included immunomodulatory and proteasome inhibitors drugs. The 25th percentile of PFS was 29 months with a median PFS almost reached (50.9 months) at 36 months (Figure 1). Ten patients (33%) relapsed during follow-up, and three patients died because of disease progression, with death occurring before ASCT in two of them. More detailed characteristics of the patients are provided in Table 1.

Of the 30 included patients, 27 could proceed to ASCT as three patients presented early signs of progressive disease with marked alteration of general condition. Among the 27 patients who underwent ASCT, 25 had consolidation chemotherapy. Post-induction WB-DW-MRI was available in 25/27 (93%) patients, of whom 22 (82%) could also receive 18-FDG PET/CT evaluation (one patient opted not to receive the post-induction FDG-PET, and there was difficulty in scheduling the exam with two other patients). Post-ASCT MRI was obtained in 27 (100%) patients, of whom 26 (96%) also had FDG PET/CT.

### 2.2. Post-Induction Chemotherapy Imaging

**Figure 2:**
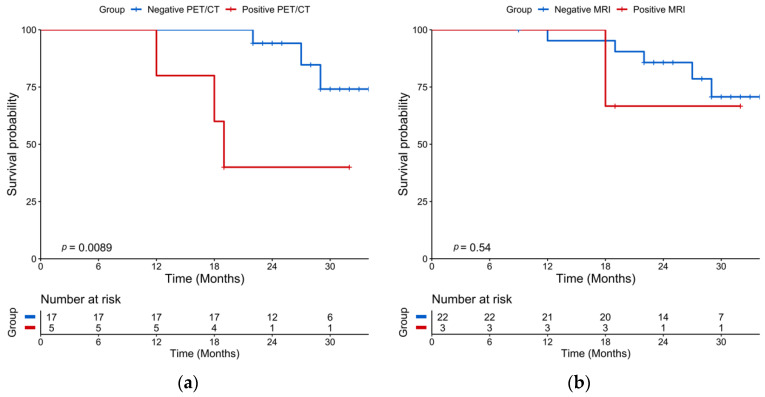
Kaplan-Meier plots of progression-free survival according to imaging performed after post-induction chemotherapy (landmarked at the end of induction chemotherapy). (**a**) 18-FDG PET/CT results; (**b**) WB-DW-MRI results.

The median time between post-induction 18-FDG PET/CT and post-induction WB-DW-MRI was ±3 days (Q1–Q3: 3–8.5 days). After induction chemotherapy, 18-FDG PET/CT was positive in 5/22 (23%) patients and negative in 17/22 (77%) patients. The Kaplan–Meier survival curves of PFS revealed a significant PFS difference between patients with a positive post-induction PET/CT with a median PFS of 19 months while the median PFS was not reached for patients with a negative post-induction PET/CT (log-rank test *p* = 0.0096) (Figure 2a). On univariate analysis, a positive 18-FDG PET/CT was associated with a shorter PFS (HR = 6.79, 95% CI 1.30-35.5, *p* = 0.02) (Table 2). Post-induction WB-DW-MRI was positive in 3/25 (12%) patients and negative in 22/25 (88%) patients, with 13/22 (59%) patients categorized as highly likely to respond and 9/22 (41%) patients as likely to respond. The difference in Kaplan–Meier survival curves of PFS by WB-DW-MRI status were not significant (*p* = 0.55) (Figure 2b). A sensitivity analysis by categorizing negative patients only as highly likely to respond and positive patients belonging to other categories was performed and did not reach significant prognostic value. Post-induction PET and MRI were concordant in 20 cases and discordant in 2 cases. There was an almost perfect agreement between the two imaging modalities (prevalence and bias-corrected kappa index: 0.82).

### 2.3. Post-ASCT Imaging

**Figure 3:**
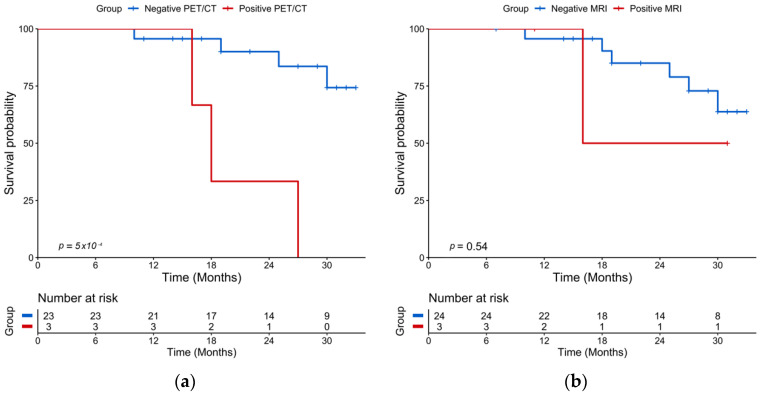
Kaplan-Meier plots of progression-free survival according to imaging performed after ASCT (landmarked at ASCT). (**a**) 18-FDG PET/CT results; (**b**) WB-DW-MRI results.

**Figure 4:**
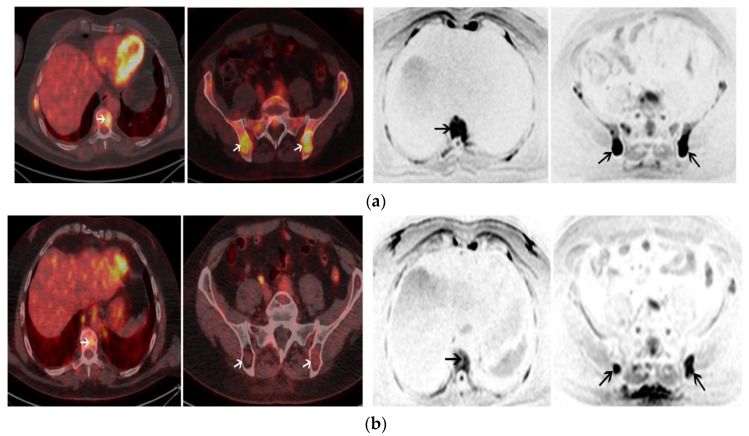
A 55-year-old male with IgA kappa MM. (**a**) Axial images of baseline 18-FDG PET/CT (left panel) shows multiple focal lesions (FL) with high uptake including one FL involving T9 and 2 FL involving the pelvis (white arrows). Axial images of baseline WB-DW-MRI (left panel) shows FL with restricted diffusion (b = 800 s/mm^2^) involving also T9 and the pelvic bone with an ADC of 920 (black arrows); (**b**) Positive post-ASCT 18-FDG PET/CT (left panel) shows a persistent uptake of T9 (FL SUVmax > liver uptake) and the regression of uptakes within FL involving the pelvis. Positive post-ASCT WB-DW-MRI (right panel) shows a regression of the restricted diffusion in T9 but the persistence of two FL in the pelvis with restricted diffusion and ≤25% increase of the ADC values (ADC = 1000). Patient was in complete remission after ASCT but relapsed 16 months after ASCT.

**Figure 5:**
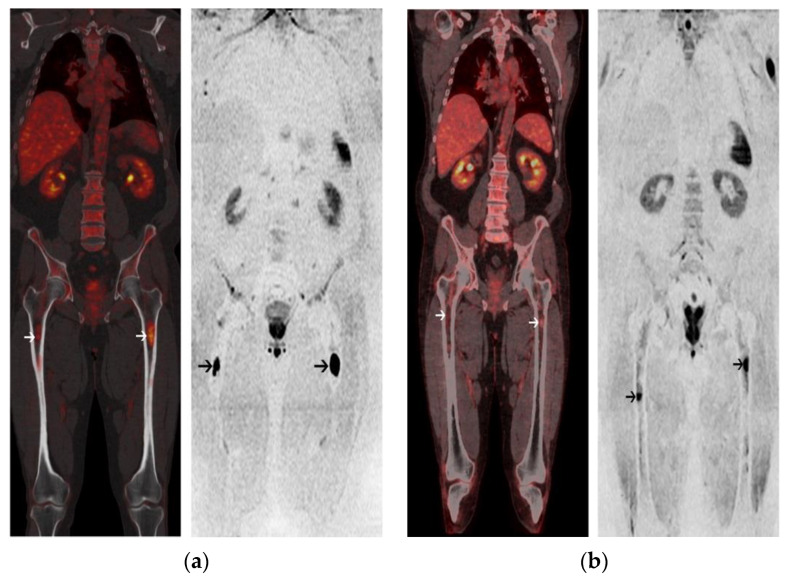
A 63-year-old male with Kappa Light Chain MM. (**a**) Coronal image of 18-FDG PET/CT at baseline (left panel) shows 2 focal lesions (FL) within the femurs (white arrows). Corresponding coronal image of baseline WB-DW-MRI (right panel) shows 2 FL with restricted diffusion within the femurs; (**b**) Post-ASCT 18-FDG PET/CT is negative and coronal image (left panel) shows a regression of uptake of the femoral FLs (FL SUVmax < liver uptake) (white arrows). Post-ASCT WB-DW-MRI shows a regression of the extent of the femoral FL (black arrows) associated with a decrease of the diffusion restriction and a significant increase of the ADC value from baseline (>40%). Patient was classified as low-risk by both imaging modalities and is in complete remission after 33 months of follow-up.

The median time between post-ASCT PET/CT and post-ASCT MRI was ±4.5 days (Q1–Q3: 3–10.8 days). After transplantation and consolidation chemotherapy, 18-FDG PET/CT was positive in 3/26 (11%) patients and negative in 23/26 (89%) patients. The median PFS was 18 months for patients with a positive PET/CT and was not reached for patients with a negative PET/CT (log rang test *p* = 0.0005) (Figure 3a). A positive PET/CT was associated with a shorter PFS on univariate analysis (*p* = 0.005) (Table 2). WB-DW-MRI was positive in 3/27 (11%) patients and negative in 24/27 (89%) patients with 17/24 (71%) patients highly likely to be responders and 7/24 (29%) likely to be responders. There was no significant association between WB-DW-MRI results and PFS (log-rank test *p* = 0.55) (Figure 3b). A sensitivity analysis by categorizing negative patients as only with highly likely to be responding and positive patients belonging to other categories was performed and did not reach significant prognostic value. Post-ASCT PET/CT and MRI were concordant in 23 cases and discordant in 4 cases. There was a substantial agreement between the two imaging modalities (prevalence and bias-corrected kappa index: 0.69). Examples of post-ASCT response assessment on 18-FDG PET/CT and WB-DW-MRI are illustrated in Figure 4 and Figure 5.

### 2.4. Baseline Imaging

18-FDG PET-CT and WB-DW-MRI were both interpreted as positive in 28/30 (93%) patients, with an almost perfect agreement between the two methods (prevalence and bias-corrected kappa index: 1.00). The following distributions of disease patterns on 18-FDG PET-CT vs. WB-DWI were found: diffuse and focal (13 vs. 17), focal (14 vs. 10), diffuse (1 vs. 1) and normal (2 vs. 2), respectively.

The prognostic influence of clinical and imaging factors at baseline are provided in Table 2. Extramedullary disease and more than four involved skeletal areas on baseline PET/CT were associated with shorter PFS.

## 3. Discussion

The evolution of MM imaging landscape with the advent of novel techniques such as 18-FDG PET/CT and WB-DW-MRI warrants the evaluation of the precise role of each of these specific newer modalities. To our knowledge, our study is the first to offer a prospective comparison of WB-DW-MRI and 18-FDG PET/CT prognostic values at two important time points of treatment response evaluation of newly diagnosed MM patients: after induction chemotherapy and after ASCT-conso.

In our study, the prognostic value of 18-FDG PET/CT was superior to that of WB-DW-MRI both after induction chemotherapy and after ASCT-conso. Our included population corresponds to current standard of care of MM patients eligible for ASCT, as all were treated with immunomodulatory drugs (IMIDs) and proteasome inhibitors (PIs) followed by a single ASCT and consolidation chemotherapy with or without maintenance [13]. Previous imaging studies have shown a good prognostic value of PET/CT performed either 7 days after the start of induction chemotherapy or after the end of induction chemotherapy [4,5,14]. In a population similar to that of our study consisting of 54 patients undergoing induction chemotherapy based on immunomodulatory drugs (IMIDs) and proteasome inhibitors (PIs), followed by ASCT, the IMAJEM study showed a trend to a significant prognostic impact of pre-transplant PET/CT on PFS [6]. Post-ASCT PET/CT was the strongest prognostic factor in our study. This is in line with previous studies from three different centers that demonstrated the ability of PET/CT to predict the outcome of patients after ASCT [4,6,15]. The liver uptake was used as a cut-off for PET/CT positivity, which is in line with the IMAJEM study [6] and was also recently pointed to as the best predictor in a study of patients enrolled in two independent European randomized phase III trials [16]. Overall, in the era of PIs and IMIDs, 18-FDG PET/CT appears to provide useful and additional information by complementing the biochemical evaluation of two important time points in the management of MM patients undergoing ASCT.

In this series, WB-DW-MRI did not reach a statistically significant impact on PFS despite standardized interpretation with the use of MY-RADS criteria [12]. This may be due to a small number of patients. However, it should be stressed that FDG-PET/CT and WB-DW-MRI criteria are fundamentally different. FDG PET/CT criteria are deemed to depict the presence or the absence of tumors, while the MY-RADS MRI criteria provide a likelihood of response but yield no information—irrespective of response—whether there is any tumor left. Performing WB-DW-MRI in addition to PET/CT did not help to identify additional non-responders as the only MRI-positive patient that relapsed was also depicted by 18-FDG PET/CT. The MY-RADS criteria that we used integrate morphological information combined with diffusion-weighted imaging in order to categorize patients’ likelihood of response, potentially lessening the important rate of false-positive findings of conventional MRI [12]. Nevertheless, in order to take full advantage of MRI in assessing response, MRI criteria need to be overhauled to more clearly describe not only how likely MM is to respond to treatment, but also how completely the tumor has been eradicated.

Previous works suggested that a rise in the ADC of MM focal lesions could be linked to cellular necrosis and biochemical response at 4 to up to 21 weeks after the start of chemotherapy [9,10,11]. However, other studies provided discrepant results [17,18]. Additionally, data regarding the impact of ADC measurements on survival were lacking. A study by Rasche et al. showed, in a mixed population of newly diagnosed MM and heavily pre-treated patients, that WB-DW-MRI read without standardized criteria could identify focal lesions with restricted diffusion after complete remission was achieved [15]. However, although fewer patients presented with residual focal lesions on 18-FDG PET/CT, its prognostic value remained superior to that of WB-DW-MRI [15]. Overall, the results of this study combined with ours seem to indicate that 18-FDG PET/CT should be the modality of choice in monitoring MM treatment response. The development and the use of novel PET/CT radiotracers should further improve the prognostication of MM patients in the next decade [19,20,21].

Some baseline factors on PET/CT are associated with PFS. Extra-medullary disease is a well-documented prognostic factor that concerned three patients in our study, with one that died before ASCT could be performed [4,5,6,22] (Table 2). The existence of >4 skeleton areas involved on 18-FDG PET/CT at baseline had a significant impact on the PFS (Table 2). This also had a negative prognostic value on baseline MRI. This highlights the impact of the tumor burden on patient prognosis. Interestingly, the presence of FL in the inferior limbs at baseline was also associated with a shorter PFS (Table 2). This is in accordance with a study that found that an appendicular skeleton involvement on CT had a pejorative prognostic value [23].

Our study had some limitations. First, the small number of patients could hamper statistical analysis with uneven proportions of patients belonging to positive imaging and negative imaging groups. Three deaths occurred, including two of them before imaging procedures for treatment response evaluation could be performed, and both these patients had extensive bone disease at baseline. Furthermore, because only one patient died after all imaging procedures were performed, we could not provide overall survival analysis.

## 4. Materials and Methods

### 4.1. Patients

From February 2017 to February 2019, patients with newly diagnosed MM eligible for ASCT were prospectively enrolled in this study. The diagnosis of MM was based on the IMWG criteria [3]. Induction chemotherapy consisted of 3 cycles, including proteasome inhibitors and immunomodulatory drugs. After induction, stem cell mobilization was followed by intensification with high-dose melphalan (200 mg/m^2^) plus ASCT and then two cycles of consolidation chemotherapy using the same molecules as for induction. The inclusion criteria included written informed consent to undergo 18-FDG PET/CT and WB-DW-MRI at diagnosis, after induction chemotherapy and after ASCT-consolidation chemotherapy (ASCT-conso). Patients were not included in the study if corticosteroid therapy was started 2 weeks prior to baseline imaging or if chemotherapy had already begun. The Ethics Committee of the District of Bordeaux approved this research. Diagnostic of relapse was made by the referee hematologist based on standard International Myeloma Working Group (IMWG) criteria [24].

### 4.2. End Points

The primary endpoint of the study was the prognostic impact of 18-FDG PET/CT and WB-DW-MRI after induction chemotherapy and after ASCT-conso.

We previously compared the diagnostic value of baseline 18-FDG PET/CT and WB-DW-MRI imaging but did not assess the prognostic value due to the short follow-up [25]. Therefore, the secondary end point of the study was the prognostic impact of baseline 18-FDG PET/CT and WB-DW-MRI. Skeletal areas were defined as previously published [25].

### 4.3. 18-FDG PET/CT

^18^F-FDG PET-CT examinations were performed on the GE-710-16S PET-CT instrument (General Electric Medical Systems, Milwaukee, WI, USA) with an injected ^18^F-FDG activity of 3 MBq/kg. Patients fasted for at least 6 h before ^18^F-FDG injection. ^18^F-FDG PET-CT was performed from the vertex to the knees, with an average total scan time of 20 min. After anonymization, ^18^F-FDG PET-CT results were reviewed on a dedicated workstation (Advantage Workstation; GE Healthcare, Chicago, IL, USA) by one experienced nuclear medicine physician (10 years of experience) blinded to the MRI, clinical and biological data.

At baseline, a Focal Lesion (FL) was defined as an area of increased uptake within the bone with a higher intensity compared with the surrounding background uptake, with or without an underlying osteolytic lesion on CT [4,6,26]. Diffuse bone marrow infiltration was defined as diffuse bone marrow uptake greater than the liver SUVmax, as described previously [6,27]. Extramedullary disease (EMD) was defined as focal uptake outside the bones that did not arise from a bone structure [6]. After induction chemotherapy and after ASCT-consolidation chemotherapy, 18-FDG PET/CT was considered negative if residual uptake (SUVmax) in FL or EMD was equal or inferior to liver SUVmax uptake, which was measured with a 4 cm ROI in the right liver, in line with the literature [6,27].

### 4.4. Whole-Body MRI with Diffusion-Weighted Imaging

WB-DW-MRI examinations were performed using a 1.5 Tesla device (Aera; Siemens Healthineers, Erlangen, Germany) with an average total scan time of 45 min. T1-weighted turbo spin echo (repetition time (TR) 785 ms; echo time (TE) 10 ms) and T2-weighted short-tau inversion recovery (TR 13,850 ms; TE 90 ms) coronal sequences were performed at the pelvis. T1-weighted turbo spin echo sequences (TR 453 ms; TE 12 ms) and T2-weighted short-tau inversion recovery (TR 7450 ms; TE 62 ms) sagittal sequences were performed at the spine. Axial diffusion-weighted sequences were acquired from vertex to knees with seven to nine stacks and b values of 50 and 800 s/mm^2^. Each stack was composed of 50 slices of 5 mm thickness (TR 7959 ms, TE 61 ms, inversion time 180 ms). Fused 3D maximal intensity projection of DW images was built.

After anonymization, WB-DW-MRI results were reviewed by one experienced radiologist with more than 20 years of experience blinded to the 18-FDG PET/CT, clinical and biological data. At baseline, an FL was defined on coronal and sagittal sequences as a lesion >5 mm with low and high signal intensity on T1- and T2-weighted imaging, respectively [6]. On DW images, an FL was defined visually as an area of focal intensity above the bone marrow background signal. Bone marrow ADC was measured at the lumbar vertebra L2 level at every FL ≥ 1 cm. Diffuse bone marrow infiltration was defined in line with the MY-RADS criteria [12].

After induction chemotherapy and after ASCT-consolidation chemotherapy, WB-DW-MRI studies were reviewed with the MY-RADS criteria [12]. Patients classified as highly responding or likely responding by MY-RADS criteria were considered with a negative MRI, whereas patients classified as with no change, likely or highly likely to be progressing were considered with a positive MRI [12].

### 4.5. Statistical Analysis

The impacts of post-induction and post-ASCT 18-FDG PET/CT and WB-DW-MRI results on Progression-Free Survival (PFS) were evaluated with landmarking methods. PFS was defined as the time from each time point (i.e., at induction chemotherapy or at ASCT) to either relapse or death. Survival curves were based on the Kaplan-Meier method and compared with the log-rank test. Cox proportional hazard models were employed to calculate the hazard ratios (HRs) of PFS predictors. Inclusion characteristics were considered as fixed covariates. Imaging and biochemical assessment of response to treatment were considered as time-dependant covariates using values at post-induction and post-ASCT time points. A two-sided *p*-value < 0.05 was considered to reflect statistical significance. All analyses were performed with the R software (ver. 3.0.2, R Foundation for Statistical Computing, Vienna, Austria).

## 5. Conclusions

In conclusion, these preliminary results from a prospective comparison of 18-FDG PET/CT and WB-DW-MRI performed at two important time points of treatment response evaluation seem to indicate that only 18-FDG PET/CT was able to predict outcomes of MM patients eligible for ASCT. Integrating PET/CT metabolic response to the overall monitoring of disease activity might be of value in further improving patient’s management in multiple myeloma.

## Figures and Tables

**Table 1:** Patients characteristics at baseline.

Characteristic	Value *N* = 30	Range or %
Median age, years (range)	56	34–66
Male	17	56
Monoclonal protein isotype		
IgG	12	40
IgA	5	17
IgD	1	3
Light chain	12	40
kappa	10	33
lambda	2	7
Low hemoglobin (≤10 g/dL)	7	63
Low platelets (<150 G/L)	3	10
High calcium (>12 mg/dL)	4	13
High LDH (>250 UI/L)	13	43
Altered renal function (creatinine ≤ 2 mg/dL)	4	13
Beta-2 microglobulin		
3.5–5.5 mg/L	7	23
>5.5 mg/L	5	17
Median bone marrow plasmocytosis, %	28	0–87
Median serum free light chain (mg/L)	343	3.2–17,600
High-risk cytogenetic: t (4;14) or del17p	2	7
ISS 1	17	57
ISS 2	8	27
ISS 3	5	17
Induction chemotherapy		
VRD	12	40
VTD	6	20
VTD-Dara	6	20
VRD-Dara	4	13
IRD	1	3
IRD-Dara	1	3

LDH: Lactate Dehydrogenase; ISS: International Staging System; VRD: Bortezomib-Lenalidomide-Dexamethasone; VTD: Bortezomib-Thalidomide-Dexamethasone; Dara: Daratumumab; IRD: Ixazomib-Lenalidomide-Dexamethasone.

**Table 2:** Univariate Analyses of Progression-Free Survival.

Variable	Hazard Ratio		
	Estimate	95% CI	*p*
Age	0.99	0.92–1.09	0.96
Male sex	2.82	0.68–11.8	0.16
Low hemoglobin (≤10 g/dL)	2.37	0.61–9.21	0.21
Low platelets (<150 G/L)	1.55	0.15–13.2	0.59
Altered renal function (creatinine ≤ 2mg/dL)	4.10	0.74–22.6	0.11
High calcium (>12 mg/dL)	1.81	0.20–16.3	0.60
High LDH (>250 UI/L)	0.85	0.21–3.37	0.82
Low albumin (<35 g/L)	2.50	0.26–24.5	0.43
High CRP (>5 mg/L)	6.30	1.26–31,6	0.03
Cytogenetic t (4;14); del17p	12.84	2.06–80.1	0.006
ISS 2 to 3	6.00	1.23–29.1	0.03
FL SUVmax > 4.2 on baseline PET/CT	2.47	0.31–19.6	0.39
FL SUVmax > 6.1 on baseline PET/CT	6.08	1.29–28.8	0.02
FL ADC on baseline WB-DW-MRI	1.00	0.99–1.00	0.58
>3FL on baseline PET/CT	2.95	0.33–0.63	0.17
>11FL on baseline PET/CT	5.06	1.28–20.0	0.02
>7FL on baseline MRI	4.38	0.23–0.87	0.07
Inferior limb involvement on baseline PET/CT	3.74	1.03–13.6	0.05
>4 skeletal areas involved on baseline PET/CT	5.46	1.49–20.0	0.01
>4 skeletal areas involved on baseline MRI	3.98	1.10–14.5	0.04
Extramedullary disease on baseline PET/CT	7.00	1.34–36.5	0.02
Diffuse disease on baseline PET/CT	1.16	0.33–4.12	0.82
Diffuse disease on baseline MRI	3.07	0.64–7.84	0.21
Response ≥ VGPR after induction chemotherapy	1.11	0.26–4.73	0.89
Response ≥ VGPR after ASCT	1.25	0.15–10.9	0.84
Positive PET/CT after induction chemotherapy	6.79	1.30–35.5	0.02
FL SUVmax > 3.95 on post-induction PET/CT	8.30	1.16–42.5	0.01
Positive PET/CT after ASCT	10.15	2.00–51.4	0.005
FL SUVmax > 3.2 on post-ASCT PET/CT	11.32	2.06–62.4	0.005
Positive WB-DW-MRI after induction chemotherapy	2.12	0.24–18.4	0.50
FL ADC on post-induction WB-DW-MRI	1.00	0.99–1.00	0.69
Positive WB-DW-MRI after ASCT	1.89	0.22–15.8	0.56
FL ADC on post-ASCT WB-DW-MRI	1.00	0.99–1.00	0.94

LDH: Lactate Dehydrogenase; CRP: C-reactive Protein; ISS: International Staging System; FL: Focal Lesion; PET/CT: Positron Emission Tomography/Computed Tomography; MRI: Magnetic Resonance Imaging; VGPR: Very Good Partial Response; ASCT: Autologous Stem Cell Transplant; WB-DW-MRI: Whole-Body Diffusion-Weighted MRI.

## Data Availability

The data presented in this study are available on request from the corresponding author.
